# Influence of error-augmentation on the dynamics of visuomotor skill acquisition: insights from proxy-process models

**DOI:** 10.1152/jn.00051.2024

**Published:** 2024-05-01

**Authors:** Pritesh N. Parmar, James L. Patton

**Affiliations:** ^1^Richard and Loan Hill Department of Biomedical Engineering, University of Illinois at Chicago, Chicago, Illinois, United States; ^2^Shirley Ryan AbilityLab (formerly The Rehabilitation Institute of Chicago), Chicago, Illinois, United States

**Keywords:** error-augmentation, intermittent feedback, multirate motor learning dynamics, proxy-process models, visuomotor skill acquisition and retention

## Abstract

Our study addresses the critical question of how learners acquire skills without the constant crutch of feedback, using a specialized training approach with intermittent feedback. Despite recognized benefits in skill retention, the underlying mechanisms of intermittent feedback in motor control neuroscience remain elusive. Leveraging a previously published dataset from visuomotor learning experiments with intermittent feedback, we tested a wide range of proxy-process models that posit the presence of an inferred error signal even when an explicit sensory performance is not present. The model structures encompassed a spectrum from first-order to higher-order variants, incorporating both constant and error-dependent rates of change in error. Furthermore, these proxy-process models investigated the impact of error-augmentation (EA) training on visuomotor learning dynamics. Rigorous cross-validation consistently identified a second-order proxy-process model structure accurately predicting motor learning across subjects and learning tasks. Model parameters elucidated the varying influences of EA settings on the rates of change in error, inter-trial variability, and steady-state performance. We then introduced a dynamic-Proxy support Multi-Rate Motor Learning (dPxMRML) model, which shed light on EA’s effects on the fast and slow learning dynamics. The dPxMRML model accurately predicted subjects’ performance during and beyond training phases, highlighting EA settings conducive to long-term retention. This research yields crucial insights for personalized training program design, applicable in neuro-rehabilitation, sports, and performance training.

**NEW & NOTEWORTHY** Breaking new ground in motor learning, our research unveils the intricacies of skill acquisition without continuous feedback. By using a specialized training approach with intermittent feedback, our study reveals the previously elusive mechanisms behind this process. The introduction of innovative proxy-process models, particularly the dynamic-Proxy support Multi-Rate Motor Learning (dPxMRML) model, brings a fresh perspective to understanding the impact of error-augmentation (EA) training on learning and retention of motor skills.

## Graphical abstract

**Figure:**
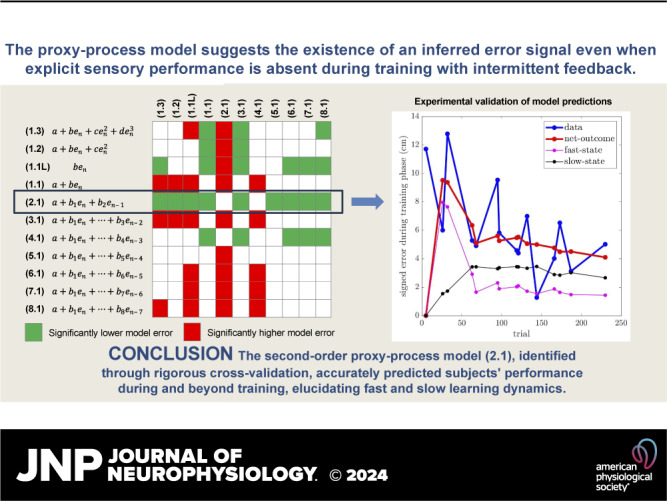

## INTRODUCTION

In motor learning, the role of performance feedback is paramount in aiding learners to assess errors and refine their movement patterns, ultimately fostering skill acquisition (1, 2). However, numerous situations arise where continuous feedback is unavailable, yet learners improve through sustained practice amidst intermittent or absent feedback (2–4). For instance, within sports training and rehabilitation therapies, coaches provide feedback only during structured sessions, leaving individuals without such guidance during their independent practice. Despite these common scenarios, existing motor learning models have yet to explore the integration of intermittent feedback into their frameworks.

Consider a simple motor learning model (5) that maintains a belief (${\hat{x}}_{n}$) about the environment to guide successful actions on trial *n* (6–8). When the predicted sensory consequences (${\hat{y}}_{n}$) of these actions do not align with the actual sensory outcomes (*y_n_*), the model updates its belief using:

(*1*) $$\begin{array}{c} {\hat{x}}_{n+1}=A\;{\hat{x}}_{n}+B\;e_{n}\\ {\hat{y}}_{n}=f\left({\hat{x}}_{n}\right)\\ e_{n}=y_{n}-{\hat{y}}_{n} \end{array}$$

Here, *A* and *B* represent retention and learning rates, respectively. Notably, $\hat{x}$ represents a hidden state inferred exclusively from feedback *y* using a function *f*. A problem arises when feedback is absent during a particular trial *n*, resulting in an inability to compute the ${\hat{x}}_{n+1}\;$ update due to the missing error term, *e_n_*.

Several strategies can be considered for addressing trials with missing feedback. These options include setting the error term (*e_n_*) to zero, omitting the trial altogether, or using data interpolation based on adjacent observations. However, adopting a zero-error approach contradicts the observed performance improvements resulting from sustained practice during feedback gaps (2–4). In addition, our previous research (9) highlights that overlooking gaps in trials fails to capture the true temporal progression of learning. Moreover, interpolating missing data may inadvertently introduce bias or unrealistic assumptions. To systematically address this challenge, a more comprehensive approach is warranted.

This approach involves generating proxy data via a process model. Specifically, a proxy estimate of error (${\hat{e}}_{n}$) can be generated as a *forward-prediction* from the observed error from previous trial (*e_n_*_−__1_) using an autoregressive model that maintains the temporal correlation of errors across all intermittent observation data. However, the functional form of the proxy-process model remains unknown. Therefore, its validity needs to be established across a diverse array of training conditions for broad applicability in generating proxy data and handling intermittent feedback scenarios.

One training condition conducive to identifying this proxy-process model is error augmentation (EA). This paradigm involves amplifying the errors made by learners during skill practice, leading to augmented feedback that reflects such manipulation of error (10). Remarkably, learners remain unaware of this feedback manipulation, yet they demonstrate distinct learning dynamics when exposed to the EA setting. These dynamics span from increased extent of learning to accelerated learning rates (10–13). Moreover, EA training incorporates occasional “catch-trials” featuring the original, unaltered training conditions. These catch-trials serve as controls, revealing how skills acquired under EA transfer back to normal conditions and offering a nuanced understanding of the effectiveness of EA (4). Consequently, EA training naturally lends itself to identifying the proxy-process model structure, as it provides both the intermittent catch-trial data and the diverse temporal error correlations within the learning curves.

In this paper, our primary objective was to discover the best proxy-process model structure for motor skill acquisition with EA. We used a dataset from our previous study (14), which comprehensively examined various forms of EA settings across eight distinct visuomotor distortion tasks, yielding multiple learning curves per subject. Drawing insights from the literature (5, 8, 15–27), we cross-validated 11 competing proxy-process model structures against these learning curves to evaluate the consistency of model predictions across subjects and tasks. These model structures encompassed a spectrum from first-order to higher-order variants, incorporating both constant and error-dependent rates of change in error. Our secondary objective was to investigate how EA settings influence the temporal progression of motor learning. This involved examining the rate of change in error, intertrial variability, and steady-state performance, guided by insights from the best proxy-process model. Our tertiary objective was to combine the best proxy-process model with a prominent multi-rate motor learning model (27) to investigate how EA settings influence the retention and rates of learning.

## MATERIALS AND METHODS

### Brief Overview of the Dataset

We used a dataset from a prior motor learning experiment (14) involving 15 right-handed healthy participants. Subjects sat in front of a planar manipulandum robot (two degrees of rotational freedom robot) and manipulated the robot’s end-effector (the handle). The position of the handle (as a cursor) and visual targets for reaching were shown using a 40-in. display, which was mounted directly above the robot and approximately centered at eye level. The display was calibrated to represent the absolute spatial workspace of the handle.

Subjects were instructed to move the cursor toward a visual target by making a quick, straight-line reach. The cursor was 2.5 mm diameter white circle, and the targets were 4.5 cm yellow “+” signs. The targets were placed at the vertices of a 15 cm equilateral triangle, and the reaching task included moving the cursor from one target to the next (target-to-target reaching). This resulted in 15 cm of ideal reach in six movement directions that were at least 60° apart. The visual location of these targets on the display was fixed for all phases and visuomotor distortions, and only the destination target for each trial was shown on the display at a time.

For each target reach, the *first ballistic launch of movement* was detected based on distance and speed thresholds (greater than 1 cm away from the start position and greater than 20 cm/s), and the end of the first ballistic launch of movement was detected based on speed threshold (less than 5 cm/s). All thresholds were calculated in the cursor space. Once the end of the first ballistic launch of movement was detected, the “+” sign for a target was changed to the 4.5 cm “x” sign. At this point, the trial was marked completed, and if the subjects had missed the target (greater than 0.5 cm away from the target position), they were instructed to navigate the cursor to the target to begin the next trial. Throughout this navigation phase of the movement, the cursor trace for the first ballistic launch of movement was displayed.

We also provided average-speed feedback of the first ballistic launch of movement using a visual bar at the bottom of the display. The subjects were instructed to match their launch speed with a reference speed bar (indicating 30 cm/s), which was drawn underneath their speed feedback bar. The initial average launch speed within 24–36 cm/s was marked satisfactory with a change in target color and speed feedback bar color to green. Blue represented slower speeds, and red represented faster speeds.

The learning tasks required adaptation to nonlinear visuomotor distortions, where cursor represented the subject’s shoulder angle versus elbow angle, instead of hand position. Angles were calculated using inverse kinematics and were mapped on to the display using eight different transformations, which resulted in eight distinct learning tasks. All subjects experienced all eight learning tasks in consecutive order, each task consisting of 250 trials, followed by a short washout phase of 30 trials.

During learning phases, the cursor position (*c_x_*, *c_y_*) was augmented (${\hat{c}}_{x},{\hat{c}}_{y}$) based on the movement errors exhibited by the subjects while reaching for the targets:

(*2*) $$\left[\begin{array}{c} {\hat{c}}_{x}\\ {\hat{c}}_{y} \end{array}\right]=\left[\begin{array}{c} c_{xd}\\ c_{yd} \end{array}\right]+{\text{EA}}_{\text{gain}}\;\left[\begin{array}{c} c_{x}-c_{xd}\\ c_{y}-c_{yd} \end{array}\right]+{\text{EA}}_{\text{offset}}\;\left[\begin{array}{c} c_{x0}-c_{xd}\\ c_{y0}-c_{yd} \end{array}\right]$$

Here, (*c_xd_*, *c_yd_*) represented the ideal straight-line path between the start and target positions of movements, (*c_x_*, *c_y_*) reflected unaugmented cursor position with visuomotor transformation specific to each learning task, (*c_x_*_0_, *c_y_*_0_) denoted the unaugmented cursor position from the initial exposure to the learning tasks (first trial). EA_gain_ indicated augmentation level for the current error (either 0, 1, 2, or 3), and EA_offset_ indicated augmentation level for error-offset (either 0, 1, or 2). Five control subjects received unaugmented cursor feedback, EA{gain 1, offset 0}, for all eight learning tasks. Other ten subjects received the augmentation, with EA{gain, offset} randomly selected for each learning task from 12 possible combinations, excluding the control combination. The EA was applied only during the first ballistic launch of movements.

The cursor positions during the first ballistic launch of movements were removed for some trials (no-vision catch trials). These no-vision trials occurred intermittently and randomly throughout the learning phases, constituting approximately one in four trials and never two in succession (totaling 74 no-vision trials out of 250 for each learning phase). To assess learning during these no-vision trials, we calculated the maximum L2-norm error between time samples of unaugmented cursor positions (*c_x_*, *x_y_*) and ideal straight-line paths between the start and target positions of movements (*c_xd_*, *c_yd_*). This error metric considered only the first ballistic launch of movements. Depending on visuomotor distortions and movement directions, deviations from the ideal path could be clockwise or counterclockwise. Thus, we assigned a positive sign to the error metric if the deviation from the ideal path was in the same direction as observed during the initial exposure to the learning tasks (first trial for each direction). Conversely, we assigned a negative sign when the deviation was in the opposite direction from what was observed during the initial exposure.

### Identification of the Proxy-Process Model Structure

We estimated the time course of visuomotor learning based on how the movement errors from no-vision trials evolved in response to various EA{gain, offset} levels. Here, our objective was to identify the relationship between the error at trial *n* + 1 (*e_n_*_+1_) and the error at trial *n* (*e_n_*):

(*3*) $$e_{n+1}=e_{n}+f\left(e_{n}\right)+\sigma dz$$

where *n* represented a consecutive trial number, *f* represented model structure, and *dz* was stochastic Wiener process that was sampled from the standard normal distribution, which was scaled using the standard deviation σ. The last term represented random effects associated with an intertrial variability in error. We considered the following first-order models with constant rates of change in error (constant with respect to error) (8, 15–17):

(*4*) $$\text{linear}:\left(1.\text{1L}\right)\;f\left(e_{n}\right)=b\;e_{n}$$

(*5*) $$\text{affine}:\left(\text{1}.\text{1}\right)\;f\left(e_{n}\right)=a+b\;e_{n}$$

We then considered the rates of change in error as linear and quadratic functions of error (5, 18–23), which led to the following first-order quadratic and cubic polynomial model structures, respectively:

(*6*) $$\text{quadratic}:\left(1.2\right)\;f\left(e_{n}\right)=a+\left(b+c\;e_{n}\right)\;e_{n}$$

(*7*) $$\text{cubic}:\left(1.3\right)\;f\left(e_{n}\right)=a+\left(b+c\;e_{n}+d\;e_{n}^{2}\right)\;e_{n}$$

We also considered the higher order model structures that included error history (24–27):

(*8*) $$k\mathrm{th}\text{-order}:\left(\text{k}.1\right)\;f\left(e_{n},\ldots ,e_{n-k+1}\right)=a+b_{1}\;e_{n}+\ldots +b_{k}\;e_{n-k+1}\;\left(2\leq k\leq 8\right)$$

### Proxy-Process Model Regression and Cross-Validation

We fitted the proxy-process models (*Eqs. 3*–*8*) to the signed error data obtained from no-vision trials of the learning curves for each movement direction, learning task, and subject. Each subject yielded 48 learning curves (six movement directions times eight learning tasks). We used the least-squares method with 1,000-fold GlobalSearch optimization (interior-point algorithm, MathWorks MATLAB R2017b). These models required an initial condition, *e*_0_, which was set at the subjects’ error during their initial exposures to the learning tasks (the first trial for each movement direction). We fitted the models without stochastic random effects (setting σ to zero), obtained the residual error, and normalized its standard deviation by the number of learning curve trials. This normalized value served as the standard deviation σ for the Wiener process in subsequent analyses.

To address potential overfitting, we conducted exhaustive cross-validation both across subjects and across learning tasks within subjects. The cross-validation across subjects was performed per each EA{gain, offset} coordinate, and subjects were partitioned into training and test sets, with a split ratio of 4/5 and 1/5, respectively. In addition, the cross-validation across learning tasks within subjects was performed only for the control subjects who received the same EA{gain 1, offset 0} for all eight learning tasks (we excluded other subjects because they received different EA coordinate per learning task). For these subjects, we partitioned the learning tasks into training and test sets with the same split ratio.

In both types of cross-validation, we exhaustively tested all possible permutations of partitioning the training and test sets. We evaluated the performance of the average model, trained on the training set and applied to the test set, using the root mean square error (RMSE). Given that the distribution of RMSE was non-normal, we computed the maximum likelihood estimate (MLE) of RMSE from kernel density estimates for each test set partition.

We performed pairwise comparisons among the models for the MLE of RMSE from all test set partitions using the left-tailed Wilcoxon signed rank test with 0.01 α level, corrected using the Bonferroni method (each model was compared against every other model; 110 total model pairs). Subsequently, we computed the model score, which represented the number of times a particular model demonstrated significantly lower RMSE compared with other models, minus the number of times other models displayed significantly lower RMSE compared with that specific model.

### Effects of EA on the Temporal Progression of Motor Learning

We exclusively examined the second-order (2.1) model to investigate the impact of EA on the temporal progression of motor learning, as revealed by its superior performance in the cross-validation analysis. For each of the second-order (2.1) model parameters (*a*, *b*_1_, *b*_2_, σ), we conducted pairwise comparisons between the control EA{gain 1, offset 0} and all other EA coordinates. These comparisons were carried out using the Wilcoxon rank sum test with a significance level of 0.05, and the results were corrected for multiple comparisons using the Bonferroni method. In addition, we individually tested each of the second-order (2.1) model parameters (*a*, *b*_1_, *b*_2_) against zero (or against one for σ) at each EA coordinate using the sign test with a significance level of 0.05. Furthermore, we applied the fitted second-order (2.1) models and computed corresponding steady-state error values at trial 250. These steady-state error values were then subjected to a sign test against zero for each EA coordinate with a significance level of 0.05.

### Effects of EA on Retention and Learning Rates

We investigated the influence of EA on retention and learning rates using a prominent multi-rate motor learning model (27), known for its insights into fast and slow learning dynamics. However, the standard model proved inadequate for handling sparse and intermittent EA datasets. To address this limitation, we developed the dynamic-Proxy support Multi-Rate Motor Learning (dPxMRML) model. This innovation combined the standard model with a second-order proxy-process (2.1) model, leveraging proxy data to support the standard model’s predictions on trials with missing observations:

(*9*) $$\begin{array}{l} \text{fast-state}:\;{\hat{x}}_{\text{f},\;n+1}=A_{\text{f}}\;{\hat{x}}_{\text{f},\;n}+B_{\text{f}}{\hat{e}}_{n}\\ \text{slow-state}:\;{\hat{x}}_{\text{s},\;n+1}=A_{\text{s}}\;{\hat{x}}_{\text{s},\;n}+B_{\text{s}}{\hat{e}}_{n}\\ {\hat{e}}_{n}=\{\begin{array}{c} \;e_{n},\text{ if available};\\ {\hat{e}}_{n-1}+f\left({\hat{e}}_{n-1},{\hat{e}}_{n-2}\right)+\sigma \;dz,\;\text{ otherwise} \end{array}\\ f\left({\hat{e}}_{n-1},{\hat{e}}_{n-2}\right)=a+b_{1}\;{\hat{e}}_{n-1}+b_{2}\;{\hat{e}}_{n-2}\\ \text{net-outcome}:{\hat{y}}_{n}={\hat{x}}_{\text{f},n}+{\hat{x}}_{\text{s},n}\\ A_{\text{s}}>A_{\text{f}};B_{\text{f}}>B_{\text{s}} \end{array}$$

Note that we specifically used the second-order (2.1) model structure due to its superior performance in the cross-validation analysis. We fitted the dPxMRML (*Eq. 9*) to the signed error data obtained from no-vision trials of the learning curves for each movement direction, learning task, and subject. We used coefficient values for the second-order (2.1) model parameters (*a*, *b*_1_, *b*_2_, σ) that were obtained from the proxy-process model regressions (Fig. 2). We used the least-squares method with 1,000-fold MultiStart optimization (interior-point algorithm, MathWorks MATLAB R2020b). The initial condition, *x*_f,0_ and *x*_s,0_, for this model was set to zero.

For each of the dPxMRML model parameters (*A*_f_, *A*_s_, *B*_f_, *B*_s_), we conducted pairwise comparisons between the control EA{gain 1, offset 0} and all other EA coordinates. These comparisons were carried out using the Wilcoxon rank sum test with a significance level of 0.05, and the results were corrected for multiple comparisons using the Bonferroni method. In addition, we individually tested the learning rates (*B*_f_, *B*_s_) against zero, the fast-retention rates (*A*_f_) against negative one and against zero, and the slow-retention rates (*A*_s_) against one and against zero at each EA coordinate using the sign test with a significance level of 0.05.

To validate the impact of slow learning dynamics of the dPxMRML model beyond the training phase, we fitted an exponential function to the error magnitudes from washout phases that followed the training. Time constants of the fitted exponential function represented rates of decay of the acquired skill during washout phases. We only analyzed EA conditions that exhibited significant non-zero slow-learning rates (i.e., EA{gain 1, offset 0}, EA{gain 2, offset 0}, EA{gain 3, offset 0}). We used the least-squares method with 1,000-fold MultiStart optimization (interior-point algorithm, MathWorks MATLAB R2020b). We conducted pairwise comparisons for the time constants between the control EA{gain 1, offset 0} and all other EA coordinates. These comparisons were carried out using the Wilcoxon rank sum test with a significance level of 0.05, and the results were corrected for multiple comparisons using the Bonferroni method.

All of the Wilcoxon rank sum tests and the sign tests were conducted using a 10^5^-fold bootstrapping approach to ensure robustness and reliability of the results. Medians and 95% confidence intervals of the median were also computed using a 10^5^-fold bootstrapping approach. Bootstrap resampling was weighted inversely based on the model regression RMSE.

## RESULTS

### Identification of the Proxy-Process Model Structure

We estimated the time course of visuomotor learning using various proxy-process models structures (*Eqs. 3*–*8*) that capture how the movement errors from no-vision trials evolved in response to various EA{gain, offset} levels. The regression statistics, as presented in Supplemental Fig. S1, revealed that the model with a greater number of parameters exhibited an increased capacity to explain variance (*R*^2^, $R_{\text{adj}}^{2}$) compared with simpler models, with the eighth-order (8.1) model demonstrating the highest performance. Notably, we observed higher maximum likelihood estimate (MLE) of $R_{\text{adj}}^{2}$ for the higher-order models in contrast to the first-order models, averaging at 91.2% and 17.7%, respectively.

**Figure 1.**
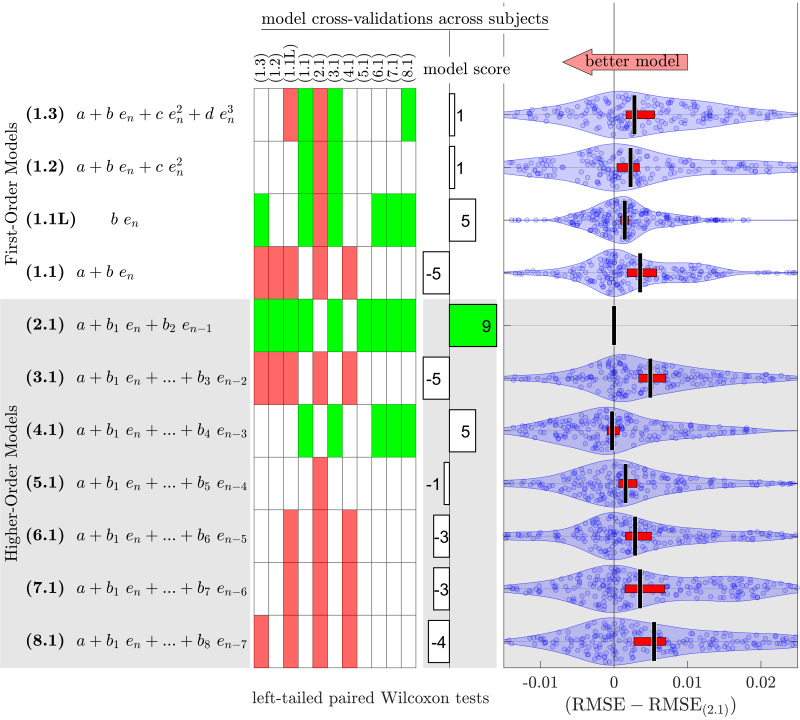
Model prediction accuracy in cross-validations across subjects, highlighting the superiority of the second-order (2.1) model. A matrix displays left-tailed paired Wilcoxon tests results, where a green square (a red square) indicates significantly lower (higher) root mean square error (RMSE) in cross-validation test set partitions (*P* < 0.01; with Bonferroni correction) for the model listed along its row compared with the model listed along its column. The model score denotes the number of times a particular model had significantly lower RMSE minus the number of times that model had significantly higher RMSE. Each blue dot represents a paired difference between its respective model’s and the second-order (2.1) model’s maximum likelihood estimate (MLE) of RMSE for a cross-validation test set partition. Jitter has been applied to the blue dots along the vertical dimension based on their kernel density estimates (shaded blue region). Vertical black lines and horizontal red bars represent medians and 95% confidence interval of the median, respectively, for the paired differences of RMSE across all cross-validation test set partitions.

To address the concern about potential overfitting, we conducted exhaustive cross-validation for all model structures. Initially, we examined the ability of each model to predict systematic changes in movement errors during no-vision trials across different subjects. The second-order (2.1) model displayed significantly lower (*P* < 0.01) root mean square error (RMSE) in the test-set partitions across subjects compared with all other models, except for the fourth-order (4.1) model (Fig. 1 and see also Supplemental Fig. S2). Among these two top-performing models, the second-order (2.1) model exhibited superior prediction accuracy, as it yielded significantly lower RMSE values (*P* < 0.01) compared with models (1.1L), (1.2), (1.3), and (5.1), a distinction that the fourth-order (4.1) model did not achieve. In addition, we assessed whether these model structures could predict systematic changes in movement errors during no-vision trials across various learning tasks within each control subject, and once again, the second-order (2.1) model emerged as the superior choice (Supplemental Fig. S3).

### Effects of EA on the Temporal Progression of Motor Learning

**Figure 2.**
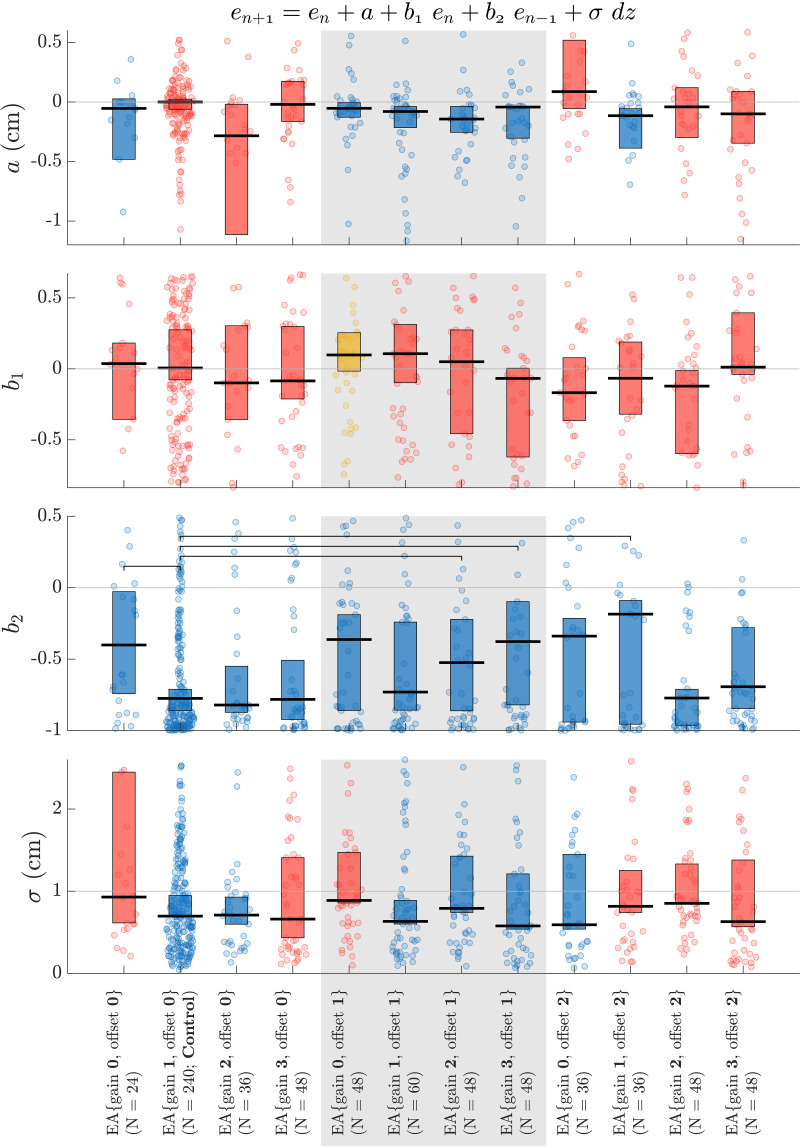
Coefficient values of the second-order (2.1) model across error-augmentation (EA) coordinates. Black lines and bars represent medians and 95% confidence intervals of the median, respectively. Each dot corresponds to a learning curve observed for a subject, task, and movement direction. Jitter has been applied to the dots along the horizontal dimension based on their kernel density estimates. Coefficient values that are significantly negative (less than one for σ) are represented in blue, while significantly positive values are shown in yellow (*P* < 0.05, sign test). Red indicates a failure to reject the null hypothesis in sign tests against zero (against one for σ) at the 5% significance level. Horizontal brackets denote significant pairwise comparisons between the control (EA{gain 1, offset 0}) and other EA coordinates (*P* < 0.05, Wilcoxon rank sum test with Bonferroni correction).

The coefficient values obtained from the second-order (2.1) model provided insights into the systematic influences of EA settings on the rate of change in error, intertrial variability, and steady-state performance (Fig. 2). Significantly negative parameter *a* values (*P* < 0.05; sign test against zero) were observed for certain augmentation settings, including EA{gain 0, offset 0}, EA{gain 0, offset 1}, EA{gain 1, offset 1}, EA{gain 2, offset 1}, EA{gain 3, offset 1}, and EA{gain 1, offset 2}. Conversely, for other EA settings (including the control), the null hypothesis regarding the sign tests against zero could not be rejected. Negative parameter *a* values indicated a consistent reduction in the rate of change in error as the learning process advanced.

In addition, significantly positive parameter *b*_1_ values (*P* < 0.05; sign test against zero) were identified for EA{gain 0, offset 1}, whereas for other EA settings (including the control), the null hypothesis for the sign tests against zero could not be rejected. Notably, all augmentation settings yielded significantly negative parameter *b*_2_ values (*P* < 0.05; sign test against zero). Furthermore, parameter *b*_2_ values for EA{gain 0, offset 0}, EA{gain 2, offset 1}, EA{gain 3, offset 1}, EA{gain 1, offset 2} were found to be significantly less negative (lower in magnitude) than the control (*P* < 0.05; pairwise Wilcoxon rank sum tests with Bonferroni correction). Positive parameter *b*_1_ values indicated increase in the rate of change in error, proportionate to the current error, whereas negative parameter *b*_2_ values indicated a reduction in the rate of change in error, proportionate to historical (previous) error, as the learning process unfolded.

The intertrial variability in error, as estimated from parameter σ values, exhibited significantly lower values than standard white noise (*P* < 0.05; sign test against one) for specific augmentation settings, such as EA{gain 1, offset 0}, EA{gain 2, offset 0}, EA{gain 1, offset 1}, EA{gain 2, offset 1}, EA{gain 3, offset 1}, and EA{gain 0, offset 2}. Conversely, for other EA settings, the null hypothesis for the sign tests against one could not be rejected. Reduced intertrial variability indicated greater consistency in the rate of change in error across practice trials.

Furthermore, the analysis revealed significantly positive steady-state errors (*P* < 0.05; sign test against zero) for EA{gain 0, offset 2}, and significantly negative steady-state errors (*P* < 0.05; sign test against zero) for specific augmentation settings, including EA{gain 0, offset 0}, EA{gain 0, offset 1}, EA{gain 1, offset 1}, EA{gain 2, offset 1}, and EA{gain 1, offset 2}. Conversely, for other EA settings (including the control), the null hypothesis for the sign tests against zero could not be rejected. Notably, steady-state errors for EA{gain 1, offset 2} were found to be significantly lower than the control (*P* < 0.05; pairwise Wilcoxon rank sum tests with Bonferroni correction). Positive steady-state errors indicated undercompensation, where the motor skill was not fully acquired through training, whereas negative steady-state errors indicated overcompensation, signifying continued learning beyond the target skill level.

### Effects of EA on Retention and Learning Rates

**Figure 3.**
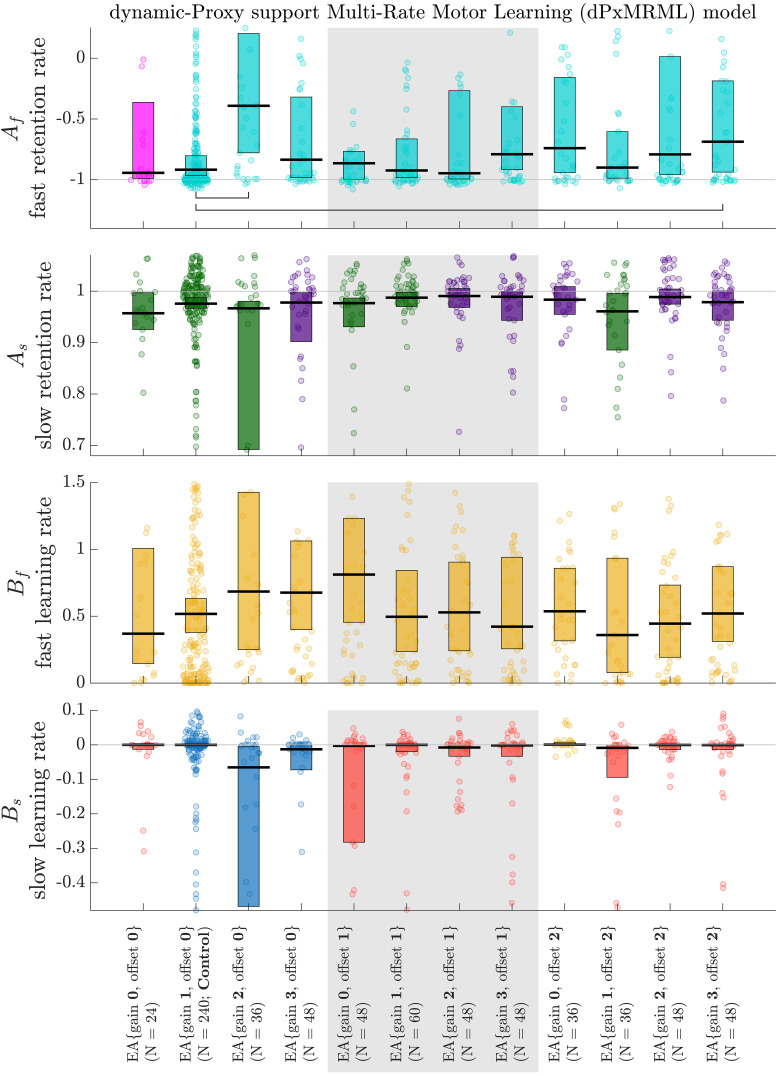
Coefficient values of the dynamic-Proxy support Multi-Rate Motor Learning (dPxMRML) model. Black lines and bars represent medians and 95% confidence intervals of the median, respectively. Each dot corresponds to a learning curve observed for a subject, task, and movement direction. Jitter has been applied to the dots along the horizontal dimension based on their kernel density estimates. Fast retention rates *A*_f_ that are significantly greater than negative one are shown in cyan (*P* < 0.05, sign test), while magenta represents a failure to reject the null hypothesis in sign tests against negative one for *A*_f_ at the 5% significance level. Slow retention rates *A*_s_ that are significantly less than one are shown in green (*P* < 0.05, sign test), while indigo represents a failure to reject the null hypothesis in sign tests against one for *A*_s_ at the 5% significance level. Coefficient values that are significantly negative are represented in blue, whereas significantly positive values are shown in yellow (*P* < 0.05, sign test). Red indicates a failure to reject the null hypothesis in sign tests against zero at the 5% significance level. Horizontal brackets denote significant pairwise comparisons between the control (EA{gain 1, offset 0}) and other EA coordinates (*P* < 0.05, Wilcoxon rank sum test with Bonferroni correction).

The coefficient values (*A*_f_, *A*_s_, *B*_f_, *B*_s_) obtained from the dPxMRML model regression provided insights into how EA settings systematically influenced retention and learning rates (Fig. 3). Notably, all augmentation settings yielded significantly positive fast-learning rates (*B*_f_; *P* < 0.05; sign test against zero). Moreover, EA{gain 1, offset 0}, EA{gain 2, offset 0}, and EA{gain 3, offset 0} exhibited significantly negative slow-learning rates (*B*_s_; *P* < 0.05; sign test against zero), whereas EA{gain 0, offset 2} displayed significantly positive slow-learning rates (*B*_s_; *P* < 0.05; sign test against 0). For the remaining EA settings for slow-learning rates, the null hypothesis for the sign tests against zero could not be rejected.

In terms of fast-retention rates (*A*_f_), all EA settings, except for EA{gain 0, offset 0}, exhibited rates falling within the range of zero to negative one (*P* < 0.05; sign test against negative one). Interestingly, EA{gain 0, offset 0} did not reject the null hypothesis for the sign tests against negative one. Furthermore, fast-retention rates for EA{gain 2, offset 0} and EA{gain 3, offset 2} were significantly less negative (lower in magnitude) than the control (*P* < 0.05; pairwise Wilcoxon rank sum tests with Bonferroni correction). As for slow-retention rates (*A*_s_), EA{gain 0, offset 0}, EA{gain 1, offset 0}, EA{gain 2, offset 0}, EA{gain 0, offset 1}, EA{gain 1, offset 1}, and EA{gain 1, offset 2} were found to be between zero and one (*A*_s_; *P* < 0.05; sign test against one). Conversely, the null hypothesis for the sign tests against one could not be rejected for the remaining EA settings.

Note that negative learning rates and retention rates (*B*_s_ and *A*_f_, respectively) arose due to the signed error metric, where the sign denoted a shift in the direction of deviations from an ideal straight-line movement. Consequently, negative rates indicate a change in the sign of model states, reflecting adaptive adjustments in motor behavior.

**Figure 4.**
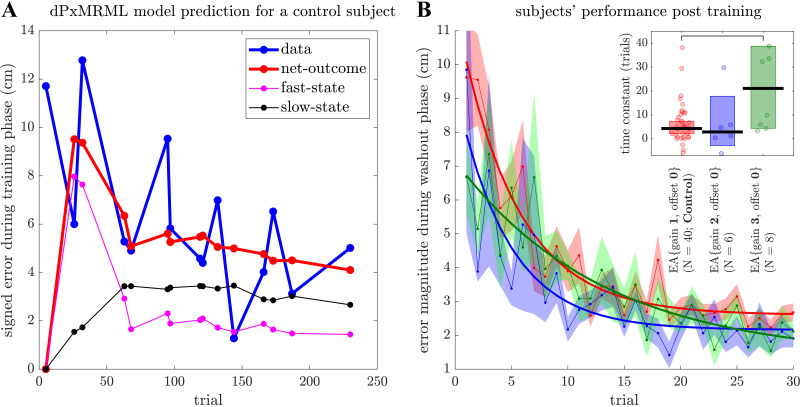
Experimental validation of dynamic-Proxy support Multi-Rate Motor Learning (dPxMRML) model predictions. *A*: an example of the dPxMRML model’s prediction of signed error (for no-vision trials) during a training phase for a control subject and a specific movement direction. *B*: performance of subjects (all trials) during washout phases following training with three different error-augmentation (EA) settings. Thin lines with shaded regions depict the averages of error magnitudes across subjects along with standard errors for each EA setting. Thick lines represent fitted exponential curves for error magnitude averages for each EA setting. *Inset*: black lines and bars represent medians and 95% confidence intervals of the median, respectively, and each dot corresponds to a subject. Jitter has been applied to the dots along the horizontal dimension based on their kernel density estimates. Horizontal brackets denote significant pairwise comparisons between the control (EA{gain 1, offset 0}) and EA{gain 3, offset 0} (*P* = 0.08, Wilcoxon rank sum test with Bonferroni correction).

The training phase predictions in Fig. 4*A* demonstrate the dPxMRML model’s maintenance of fast and slow learning states, enabling accurate forecasts of the signed error for the next trial based on the proxy-process generated error-estimate for the current trial. As the training phase progressed, the model adapted and refined its predictions, gradually improving its accuracy despite intermittent data observations.

The examination of slow learning dynamics in the dPxMRML model provides valuable insights into the persisting impact of acquired skill beyond the training phase. Variations in slow-retention rates observed across different EA settings underscore the significant influence of EA conditions on the long-term retention of skills. To validate this implication, we fitted an exponential function to the error magnitudes observed during washout phases that followed the training. The results of this analysis confirmed our model’s predictions (Fig. 4*B*). Specifically, among the EA settings with non-zero slow-learning rates, EA{gain 3, offset 0}, exhibiting higher slow-retention rates, displayed longer time-constants during washout phases compared with the control (21.1 median trials vs. 4.34 median trials for the control; *P* = 0.08; pairwise Wilcoxon rank sum tests with Bonferroni correction). Conversely, EA{gain 2, offset 0}, with lower slow-retention rates, demonstrated shorter time-constants in the washout phases compared with the control (2.90 median trials vs. 4.34 median trials for the control; *P* = 0.51; pairwise Wilcoxon rank sum tests with Bonferroni correction). These findings strongly support the idea that EA settings with higher slow-retention rates indeed lead to prolonged retention of learned motor skills, whereas settings with the lower rates lead to quicker skill deterioration.

## DISCUSSION

This study investigated the effects of error augmentation (EA) on the time course of visuomotor learning, using proxy-process models. Through extensive cross-validation of various model structures, we found that the second-order (2.1) proxy-process model systematically was able to predict the observed time courses of motor learning across subjects and learning tasks (Fig. 1, Supplemental Figs. S2 and S3). In addition, the second-order model parameters (*a*, *b*_1_, *b*_2_, σ) revealed the varying influences of EA settings on the rate of change in error, intertrial variability, and steady-state performance (Fig. 2). Moreover, we introduced dynamic-Proxy support Multi-Rate Motor Learning (dPxMRML) model, a fusion of the standard multi-rate motor learning model with a proxy-process model, shedding light on the varying effects of EA settings on the fast and slow learning dynamics (Fig. 3). Importantly, the dPxMRML model demonstrated remarkable accuracy in predicting subjects’ performance both during and beyond the training phases in the presence of intermittent observation data (Fig. 4).

Using an analogous first-order (1.1) model, we successfully replicated the findings of a prior study (10) concerning the impact of EA on the time constant and extent of learning (Supplemental Fig. S4), but our analysis provides new interpretations. Similar to their results (10), we observed significantly faster rates of change in error (parameter *b*) for EA{gain 2, offset 0} compared with the control (*P* < 0.05; pairwise Wilcoxon rank sum tests with Bonferroni correction) and comparable rates of change in error (parameter *b*) for EA{gain 1, offset 1} versus the control. We also observed greater extent of learning for EA{gain 1, offset 1} (significantly negative parameter *a* values; *P* < 0.05; sign test against zero) compared with the control (the null hypothesis regarding the sign tests against zero could not be rejected). Furthermore, our study extended its scope by examining various other combinations of EA-gain and EA-offset levels, allowing for the signed error metric, and exploring alternative model structures suggested by the literature. Collectively, our results suggest that the discrete second-order (2.1) model offers a more accurate description of the time course of motor learning than their continuous exponential model (10).

One compelling reason for the superiority of a second-order (2.1) model in capturing the learning process lies in its capacity to facilitate robust error processing. Motor learning necessitates not only the acquisition of error information but also the ability to discern the consistency of this error amidst motor and sensory noise and changing environmental conditions (28). Previous research has convincingly demonstrated the detectability of motor learning even in uncertain environments (15, 29, 30). Moreover, insights from sensorimotor adaptation studies suggest that the brain optimizes performance when confronted with noise and ambiguity, aligning with Bayesian processing principles (31–33). However, Bayesian frameworks require probability estimates for the task (prior) and sensory feedback (likelihood). Constructing these probabilities from a single event is impractical; instead, they are refined through a history of experiences. The challenge lies in determining how many past experiences yield reliable error signals for synthesizing these probabilities. Memorizing all past experiences can be resource-intensive for the nervous system, with older experiences potentially holding little relevance to current tasks. A second-order process, strongly supported by our findings in this study, retains memory of the past two experiences and offers a persistent error component across trials. Similar second-order processes have been identified in various contexts, including multiple time scales in learning and forgetting curves (34–36), implicit and explicit processes driven by prediction and target error (37, 38), and as a two-rate state-space model for describing force-field adaptation (27).

In addition to the persistent error, knowledge of results appears to be a crucial factor in facilitating effective motor learning. Our study used two types of EA conditions: EA-gain provided real-time visual feedback of current hand movement (closed-loop), whereas EA-offset offered visual feedback based on prerecorded initial exposure movement (open-loop). Notably, the EA-offset condition alone did not provide knowledge of results. When EA-gain remained at zero (EA{gain 0, offset 0}, EA{gain 0, offset 1}, and EA{gain 0, offset 2}), the feedback did not reflect changes associated with motor learning, resulting in slow rates of change in error (parameter *b*_2_) and high intertrial variability (parameter σ). However, increasing EA-gain to 1 (EA{gain 1, offset 0}) and then to 2 (EA{gain 2, offset 0}) resulted in feedback that accurately reflected changes tied to learning, leading to faster rates of change in error and lower intertrial variability. This aligns with findings from the motor learning literature, which highlight that increased availability of information feedback enhances the rate of performance improvement over trials (4, 39, 40). Moreover, EA-gain not only amplifies error magnitude but also magnifies perceived error variability, resembling action exploration in reinforcement learning architectures (41). Consequently, these learning enhancements may stem from the subjects’ heightened perception of action exploration, enabling them to acquire more comprehensive knowledge about the task (42).

The timing and frequency of feedback also play pivotal roles in the process of motor learning. The preference for intermittent and sparse feedback over continuous feedback in motor learning has been well established (2, 4). As too much or too little feedback can hinder effective skill acquisition, striking the right balance is essential. Although continuous feedback offers immediate corrections and guidance, it often unintentionally fosters dependency on external cues, impeding the development of learners’ intrinsic error-detection and correction mechanisms (28). In contrast, intermittent and sparse feedback strategies encourage active engagement, self-assessment, and problem-solving, empowering individuals to independently identify and rectify errors. Although the significance of feedback frequency in motor learning has been widely discussed in the literature (43), the learning process during intermittent feedback has not been comprehensively modeled until now. In this study, we introduced the dPxMRML model, an innovative computational framework designed to simulate the learning process in the presence of intermittent feedback. This model was rigorously validated using experimental data. This model contributes significantly to the field as it enables the design of effective feedback schedules.

Concurrent practice of multiple tasks naturally leads to sparse feedback, presenting a distinctive challenge in designing effective task schedules, a crucial factor impacting skill acquisition and retention (43). For instance, when practicing reaching in multiple different directions under the same visuomotor distortion, there is a lack of feedback for unpracticed directions during a trial. In a simulation study (44), researchers demonstrated optimal scheduling for two tasks based on their learning difficulties to maximize overall long-term retention. They used a learning model with two slow processes (one for each task) and a shared fast process, and they assumed zero error for the slow-state specific to a task on a given trial when it was not actively practiced (44). In contrast, our dPxMRML model offers a fresh perspective by incorporating both a fast and a slow process for each movement direction [similar to the MOSAIC model (8)] and allows for the utilization of proxy data when direct feedback about a specific movement direction is unavailable. It remains to be seen whether their model (44) or the dPxMRML model offers the best task schedules (the selection of movement directions on a trial-by-trial basis), having superior validation accuracy and improved long-term retention.

We exclusively used no-vision trials to isolate changes in the internal model (6–8), as trials involving vision (feedback) might exhibit different behaviors due to strategy adaptation or feedback corrections. In case behavior is contaminated by feedback, one can analyze the very beginning part of the movement. Researchers have reported latencies of ∼150 ms for any visuomotor feedback correction in movements (45), and thus we initially tried error calculated from the first 150 ms of the movements from both vision and no-vision trials. However, this error metric yielded a notably low signal-to-noise ratio due to its reliance on a small movement segment. Moreover, these initial movement segments were very close to ideal straight-line paths, resulting in frequent sign changes that primarily reflected natural motor variability rather than intended movements. Consequently, in this study, we opted to utilize the maximum error observed within the first ballistic launch of movements exclusively from the no-vision trials (duration of 740.6 ± 87.8 ms; means ± SD across subjects). This choice offered a better signal-to-noise ratio and more reliable detection of error sign.

Although the second-order (2.1) model accounted for a significant portion of the variance in the learning data (MLE *R*^2^ of 88% and median *R*^2^ of 64%, Supplemental Fig. S1) and performed well in cross-validations, it occasionally exhibited oscillations during the trial gaps of intermittent no-vision catch trials. These oscillations may be attributed to the higher EA-gain potentially inducing frequent changes in launch angles (resulting in clockwise or counterclockwise deviations) during reaching along an ideal straight-line path, corresponding to changes in the sign of error. It is important to note that we could not confirm this hypothesis due to the lack of consecutive no-vision trials in our dataset. Alternatively, these oscillations might not have biological relevance and could be a result of numerical conditioning issues. Some combinations of model parameters (*b*_1_ and *b*_2_) can lead to imaginary eigenvalues, and since the model regression was unconstrained, such oscillations may arise. Despite this limitation, the proxy-process model captured the overall trend of the time course of motor learning, even in the presence of occasional oscillations.

The suboptimal model fits in our study, as indicated by the interquartile range of *R*^2^ spanning from 45% to 85% for the second-order (2.1) model (Supplemental Fig. S1), can be primarily attributed to a low signal-to-noise ratio (SNR). This low SNR arises from the unique challenges posed by the EA experimental conditions, the inherent difficulty of the learning tasks, and the randomized experimental design. Notably, we identified negative correlations between *R*^2^ and standard deviation of residual error for the second-order (2.1) model across many EA settings (Supplemental Fig. S5), highlighting that low SNR, approximated as a high standard deviation of residual error, adversely affected the model fit quality (*R*^2^). Furthermore, the visuomotor distortions utilized for training were characterized by nonlinearity and a high level of difficulty in the learning process. The movement traces during the initial training trials, particularly the corrective actions following the first ballistic launch, appeared random and prolonged. Similar outcomes have been reported in other studies using comparable visuomotor distortions (46). In addition to the task complexity, our experimental design incorporated a randomized trial sequence across movement directions. It is essential to note that the models evaluated in this study exclusively considered the first ballistic movements and did not account for any learning that might have occurred during the feedback correction phase, when subjects navigated toward the target after the first ballistic launch. These factors combined likely contributed to the observed low SNR.

Generalization is an important aspect of motor learning, where motor commands reinforced in one context are exhibited in another (47–52). However, in this study, we did not use any model structure that can account for generalization across movement directions because a separate analysis (53) revealed that the model coefficient that controlled for generalization of error from one movement direction to neighboring movement directions (60° and more of angular separations) was only 11% of the model coefficient that maintained direction-specific error and did not generalize. Similarly, others have also provided evidence for narrowly generalizing and spatially local learning where errors are perceived (17, 54–59).

Although exposure to conflicting visuomotor rotations or force fields is known to be challenging and susceptible to interference (27, 60–65), our study avoided intermixing different visuomotor distortions (tasks) but instead presented them sequentially with short washout periods. This sequential approach raised concerns about potential interference from previous exposures affecting subsequent learning. However, as we maintained a consistent order of task presentation for all subjects, any task-related interference effects would be comparable across subjects. Importantly, our analysis did not reveal such comparable interference across subjects.

In addition to its implications for motor learning, our proposed proxy-process model holds considerable neurological significance. The model provides an internal representation of body-machine or body-environment interactions, allowing for the generation of predictions about the outcomes of these interactions based on simulations. The proxy-process model does not seek to establish a causal parametric relationship, as seen in forward models, or an inverse model that decodes actions into specific consequences. Instead, the proxy-process model captures the correlation between different states of interaction. In situations where feedback is intermittently absent, these internally simulated predictions can function as valuable proxies or substitutions, contributing to adaptive behavior. This nuanced perspective on internal representation of interactions sheds light on how the nervous system may navigate scenarios with gaps in feedback, offering insights into the cognitive processes that underlie adaptive behavior. Further exploration of the neurological underpinnings of proxy-process models may unveil novel aspects of information processing in the brain, contributing to our broader understanding of cognitive functions beyond the scope of motor learning.

In conclusion, our investigation examined the influence of error-feedback and its augmentation on training outcomes, consistently revealing a second-order structure in motor learning across different EA methods and highlighting the critical role of EA{gain, offset} levels in shaping adaptation rates and accuracy. The dPxMRML model proved instrumental in understanding the dynamics of the fast and slow learning processes in the presence of sparse observations. This model offers a versatile framework for assessing and optimizing learning, emphasizing the importance of training conditions in enhancing skill retention and long-term performance.

Our findings carry broader implications for designing personalized training programs. As human-machine interactions continue to evolve, our work contributes to establishing scientific frameworks for identifying optimal training conditions (66). Although our study provides a foundational understanding of motor learning, we acknowledge the complexity of extrapolating findings to more intricate movement tasks (i.e., from simple two-dimensional movements to more complex three-dimensional movements). Nevertheless, our research could contribute to developing tailored training programs in the fields such as neuro-rehabilitation, sports, piloting, and performance training. In addition, the innovative learning models introduced hold promise for informing advancements in artificial intelligence, facilitating the development of machine intelligence grounded in the principles of nervous system processing.

## DATA AVAILABILITY

The data that support the findings of the present article are available from the corresponding author upon reasonable request.

## SUPPLEMENTAL DATA

10.6084/m9.figshare.25696257.v1 Supplemental Figs. S1–S5: https://doi.org/10.6084/m9.figshare.25696257.v1.

## GRANTS

The research reported in this publication was supported by the National Institutes of Health under Award Numbers F31-NS100520 and 2R01-NS053606.

## DISCLOSURES

No conflicts of interest, financial or otherwise, are declared by the authors.

## AUTHOR CONTRIBUTIONS

P.N.P. and J.L.P. conceived and designed research; P.N.P. performed experiments; P.N.P. analyzed data; P.N.P. and J.L.P. interpreted results of experiments; P.N.P. prepared figures; P.N.P. drafted manuscript; P.N.P. and J.L.P. edited and revised manuscript; P.N.P. and J.L.P. approved final version of manuscript.
